# Fatal Aortic Rupture at Term Pregnancy Caused by Vascular Ehlers–Danlos Syndrome Diagnosed by Postmortem Genetic Testing Using Formalin‐Fixed, Paraffin‐Embedded Tissue

**DOI:** 10.1111/jog.70299

**Published:** 2026-05-06

**Authors:** Mari Tadakawa, Tomomi Yamaguchi, Hasumi Tomita, Noriyoshi Mochii, Hirotaka Hamada, Mari Tsubata, Tomoki Kosho, Yoko Aoki, Masatoshi Saito

**Affiliations:** ^1^ Department of Obstetrics and Gynecology Tohoku University Graduate School of Medicine Sendai Miyagi Japan; ^2^ Center for Medical Genetics, Shinshu University Hospital Matsumoto Japan; ^3^ Department of Medical Genetics Shinshu University School of Medicine Matsumoto Japan; ^4^ Division of Clinical Sequencing Shinshu University School of Medicine Matsumoto Japan; ^5^ Department of Medical Genetics Tohoku University Graduate School of Medicine Sendai Miyagi Japan; ^6^ Research Center for Supports to Advanced Science, Shinshu University Matsumoto Japan; ^7^ BioBank Shinshu, Shinshu University Hospital Matsumoto Japan

**Keywords:** *COL3A1*, maternal death, next‐generation sequencing, postmortem genetic testing, vascular Ehlers–Danlos syndrome

## Abstract

Vascular Ehlers–Danlos syndrome (vEDS) is a life‐threatening connective tissue disorder that often remains undiagnosed before pregnancy and carries a markedly high risk of maternal mortality. We report the case of a 34‐year‐old pregnant woman who experienced sudden abdominal pain at 39 weeks of gestation and died shortly after delivery. Autopsy revealed an aortic rupture with histopathological findings suggestive of vEDS. Her family history included her father's sudden vascular death, and her personal history was notable for easy bruising and early‐onset varicose veins. Next‐generation sequencing‐based postmortem genetic testing (PMGT) using formalin‐fixed, paraffin‐embedded liver tissue confirmed a pathogenic variant in *COL3A1*. This result facilitated genetic counseling for the family, allowing presymptomatic diagnosis and preventive management, including celiprolol therapy for at‐risk relatives. This case underscores the value of PMGT in identifying the underlying cause of unexpected maternal death, particularly when conventional samples are unavailable.

## 1 Introduction

Aortic rupture remains one of the most common causes of maternal death during pregnancy [1]. Unfortunately, among women who die from aortic dissection or rupture during pregnancy, most were not previously diagnosed or recognized as being at‐risk, even when a family history of vascular disease was present [1]. Postmortem genetic testing (PMGT) plays a crucial role in identifying the cause of death and provides important implications for surviving family members [2]; however, it is still rarely performed in perinatal cases.

In this report, we describe a 34‐year‐old pregnant woman who developed an aortic rupture at 39 weeks of gestation, which led to her death. Her family history and personal clinical history, along with the pathological findings from the autopsy, raised suspicion of hereditary connective tissue disorders such as vascular Ehlers–Danlos syndrome (vEDS). The family strongly wished to confirm the diagnosis; however, no blood samples or other tissues were available except for formalin‐fixed, paraffin‐embedded (FFPE) samples. Therefore, we performed next‐generation sequencing‐based PMGT using DNA extracted from FFPE liver tissue, which confirmed a pathogenic variant in *COL3A1*. Importantly, this case highlights several clinically relevant issues, including the difficulty of recognizing vEDS during pregnancy, the feasibility of PMGT using FFPE tissue, and the clinical implications of establishing a genetic diagnosis for surviving family members.

## 2 Case Presentation

A 34‐year‐old woman, gravida 1, para 0, presented with sudden‐onset abdominal pain at 39 weeks of gestation. She had a medical history of Graves' disease. She also had varicose veins and had consulted a vascular surgeon during her first trimester, but the findings were considered within the normal range. The abdominal pain was continuous and severe, and her husband noticed that she had lost consciousness; therefore, she was transferred to the obstetrics ward by ambulance. On examination, she was in severe hypovolemic shock with intense abdominal pain, and fetal heart rate monitoring revealed recurrent, prolonged decelerations to 60 beats per minute. The obstetric team decided to perform an emergency cesarean section. Two hours after the onset of abdominal pain, a live male infant weighing 2878 g was promptly delivered and transported to the neonatal intensive care unit for respiratory distress. The Apgar scores were 5 and 8 at 1 and 5 min, respectively, with an umbilical cord arterial pH of 7.057. He underwent brain hypothermia therapy for possible neonatal asphyxia and was discharged on day 42 of life.

**FIGURE 1:**
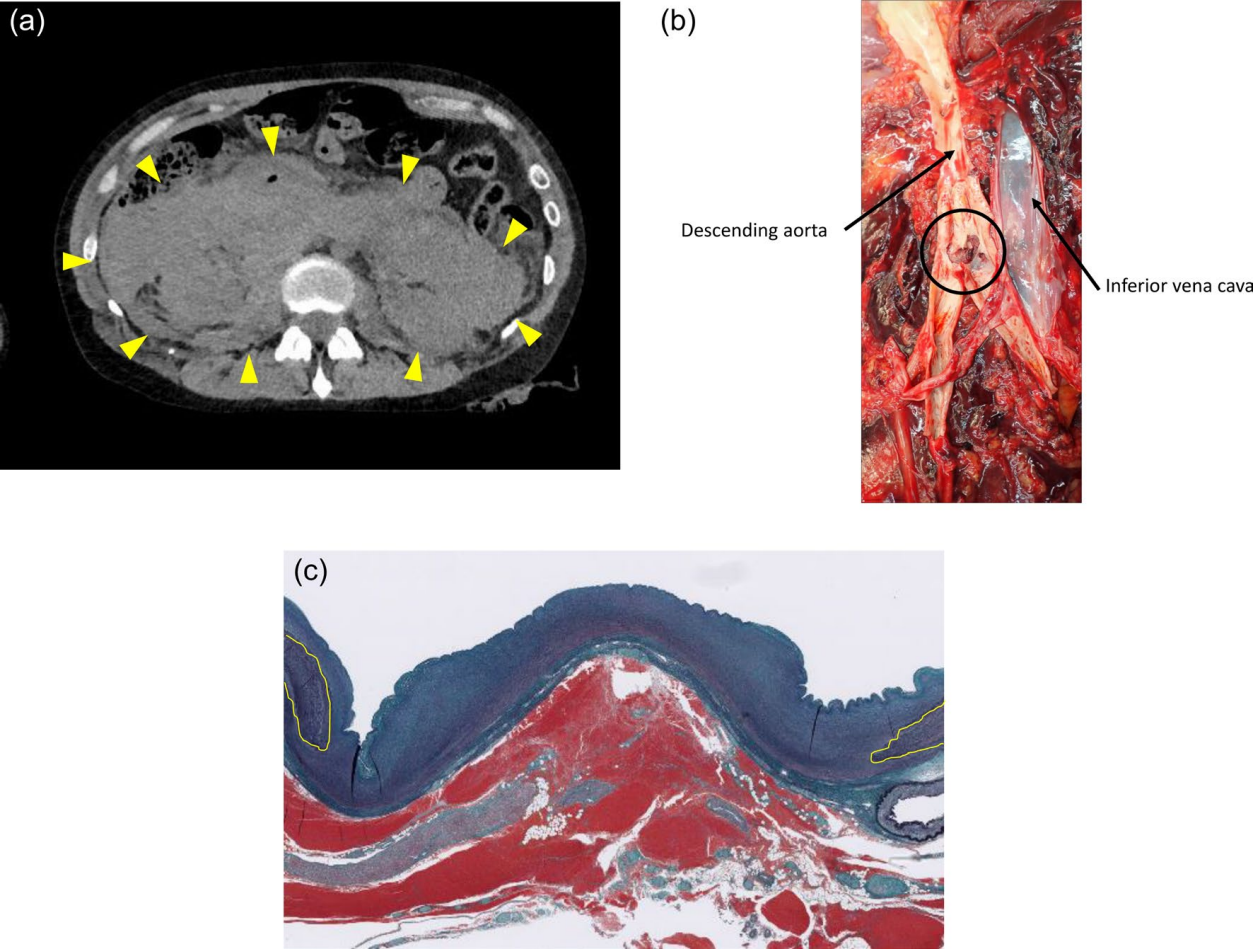
(a) Postmortem computed tomography showing a large right‐sided retroperitoneal hematoma. (b) Pathological autopsy revealing a 5 mm longitudinal friable tear in the descending aorta, located above the bifurcation of the common iliac arteries. (c) Elastica‐Masson stain of the descending aortic wall showing partial loss of elastic fibers (yellow area indicates elastic fibers).

During the cesarean section, the obstetric team noted a large hematoma in the right retroperitoneal space. They performed gauze packing and initiated blood transfusion while preparing for ambulance transfer to our hospital, a major perinatal medical center in the region. Before entering the ambulance, her condition deteriorated, leading to cardiopulmonary arrest. She was transferred to our hospital three hours after the onset of abdominal pain. We performed an emergency laparotomy to control the bleeding, accompanied by massive blood transfusion and cardiopulmonary resuscitation; however, she could not be resuscitated. Postmortem computed tomography revealed a large right‐sided retroperitoneal hematoma (Figure 1a).

**FIGURE 2:**
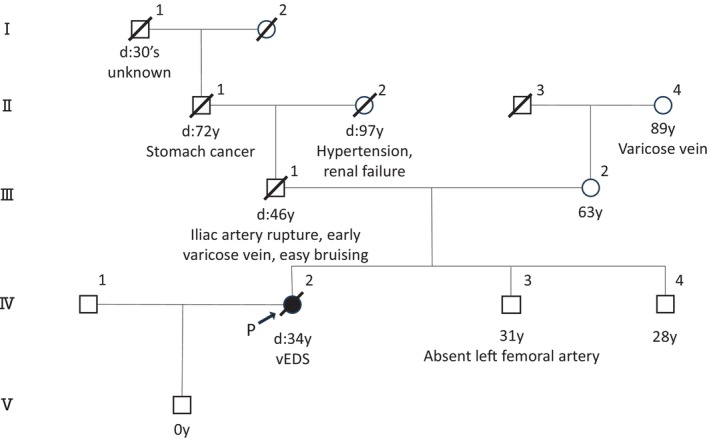
Family pedigree. None of the family members had been diagnosed with vEDS. The patient's father died at 46 years of age due to rupture of the left iliac artery and had a history of early‐onset varicose veins and easy bruising.

With the consent of the bereaved family, an autopsy was conducted and revealed a 5 mm longitudinal friable tear in the descending aorta (Figure 1b). Histopathological examination demonstrated a loss of elastic fibers and type III collagen in the aortic wall (Figure 1c). Furthermore, her family history revealed that her father had died at 46 years of age from spontaneous rupture of the left iliac artery (Figure 2). According to information provided by the family, the patient had a history of easy bruising and early‐onset varicose veins. These findings, together with the arterial rupture and the family history of arterial rupture at a young age, were considered consistent with the major and minor clinical features described in the diagnostic criteria for vEDS. Given that she fulfilled the clinical diagnostic criteria of vEDS, we provided genetic counseling to her family. As a result of this counseling, the family also strongly requested PMGT, as they were concerned about the possible inheritance of the condition by her infant.

With the consent of the patient's family and approval by the institutional ethics committees (Shinshu University School of Medicine [#6070]; Tohoku University Graduate School of Medicine [#2019–1‐776]), PMGT was conducted to confirm the suspected diagnosis of vEDS. DNA was extracted from FFPE tissue samples obtained during the pathological autopsy. The extraction process was performed using the Cobas DNA Sample Preparation Kit (Roche Molecular Systems) in accordance with the manufacturer's protocol.

Targeted NGS for the *COL3A1* gene—the gene most commonly implicated in vEDS—was conducted on an Ion GeneStudio S5 system (Thermo Fisher Scientific) using a custom‐designed panel optimized for FFPE samples. Uracil‐DNA glycosylase treatment was applied before sequencing to remove uracil residues formed by cytosine deamination. The detected variants were annotated using SnpEff and SnpSift. A previously reported heterozygous variant, NM_000090.3:c.2168G>A (p.Gly723Asp), was identified [3]. Comprehensive genetic counseling was provided to the family both before and after the analysis.

## 3 Discussion

vEDS is a rare autosomal dominant disorder most commonly caused by pathogenic variants in the *COL3A1* gene, which encodes type III procollagen [4]. Complications resulting from decreased tissue integrity are often life‐threatening and include spontaneous rupture of arteries, the intestinal tract, and other organs [5, 6]. Diagnosis is primarily clinical and is based on major findings such as arterial rupture at a young age, spontaneous intestinal rupture, or uterine rupture during pregnancy, as well as characteristic features including thin, translucent skin, easy bruising, and distinctive facial appearance [7].

Pregnancy significantly increases the risk of catastrophic vascular events in women with vEDS, with most fatal events occurring in the peripartum or postpartum period [5]. The maternal mortality rate associated with vEDS‐complicated pregnancies has been reported to be approximately 5% per pregnancy [5, 6, 7]. Early recognition of suggestive clinical features and family history may allow stratification and proactive management during pregnancy [8]. However, as illustrated in this case, such opportunities are sometimes missed, and catastrophic vascular events may be the first manifestation. In such circumstances, PMGT may become the only means to establish a definitive diagnosis and to protect surviving family members.

Recently, PMGT has been increasingly utilized in death investigations, particularly in cases where family history and clinical features suggest an autosomal dominant heritable disorder [2]. In cases of sudden cardiac death, PMGT has identified pathogenic or likely pathogenic variants in approximately 15% of patients [9], enabling screening and potential preventive interventions for at‐risk relatives. Similarly, PMGT is valuable for elucidating the underlying causes of sudden maternal deaths; however, reports describing its use in perinatal cases remain extremely limited.

In cases of maternal death, PMGT provides critical information for surviving family members, including newborns, thereby enabling presymptomatic diagnosis and preventive strategies. For example, Björck et al. [10] reported a case of PMGT in a maternal death that occurred 9 days after delivery, which revealed a pathogenic variant in the *COL3A1* gene and subsequently allowed early diagnosis and celiprolol management of the affected child. This highlights that PMGT is not merely confirmatory but can substantially alter the clinical trajectory of surviving family members.

However, the need for genetic evaluation is not always recognized immediately after death, particularly when families are still coping with the acute loss. In such situations, recognizing that genetic analysis can be performed using DNA extracted from FFPE tissue is important, as it allows postmortem diagnosis even when conventional genetic samples are unavailable [11]. In the present case, PMGT using DNA extracted from FFPE liver tissue successfully identified a pathogenic variant in *COL3A1*.

This case provides several important clinical lessons. First, subtle clinical features such as easy bruising, early‐onset varicose veins, and a family history of vascular rupture may provide important clues for the diagnosis of vEDS during pregnancy. Second, PMGT using DNA extracted from FFPE tissue can be successfully applied even when conventional samples such as blood are unavailable. Third, establishing a genetic diagnosis after maternal death may have immediate clinical implications for surviving family members, particularly newborns who may benefit from early surveillance and preventive therapy.

In conclusion, this case highlights the critical role of PMGT in identifying underlying hereditary disorders after maternal death, supporting transparent communication with families, and ensuring timely, appropriate care for at‐risk relatives. When conventional genetic samples are unavailable, analysis using DNA extracted from FFPE tissue may provide a practical alternative for establishing a molecular diagnosis. Continued research and multidisciplinary collaboration among obstetricians, pathologists, and clinical geneticists are necessary to optimize the management of vEDS during pregnancy and to facilitate the integration of genetic testing into standard maternal mortality investigations.

## Author Contributions

**Mari Tadakawa:** writing – original draft, data curation, visualization. **Tomomi Yamaguchi:** writing – original draft, investigation. **Noriyoshi Mochii:** investigation. **Hirotaka Hamada:** writing – review and editing, investigation. **Hasumi Tomita:** writing – review and editing, project administration. **Mari Tsubata:** investigation. **Tomoki Kosho:** writing – review and editing, project administration. **Yoko Aoki:** investigatin, project administration. **Masatoshi Saito:** supervision.

## Disclosure

An earlier version of this manuscript was presented at the 60th annual meeting of the Japan Society of Perinatal and Neonatal Medicine, which was held in Osaka, Japan, on 13 to 15 July 2024.

## Ethics Statement

This study was approved by the institutional ethics committees (Shinshu University School of Medicine [#6070]; Tohoku University Graduate School of Medicine [#2019–1‐776]).

## Consent

Informed consent for publication of this case report and the accompanying images was obtained from the patient's next of kin.

## Conflicts of Interest

Tomomi Yamaguchi and Tomoki Kosho serve as endowed chairs of the Division of Clinical Sequencing, Shinshu University School of Medicine, which is sponsored by BML Inc. and Life Technologies Japan Ltd., a subsidiary of Thermo Fisher Scientific Inc.

## Data Availability

The data supporting the findings of this study are not publicly available due to ethical and privacy restrictions, as they contain potentially identifiable patient information, including genetic data. The study was approved by the institutional ethics committees (Shinshu University School of Medicine [#6070]; Tohoku University Graduate School of Medicine [#2019‐1‐776]), which impose restrictions on data sharing to protect patient confidentiality. Data access may be considered upon reasonable request to the corresponding author, subject to approval by the institutional ethics committees and in accordance with institutional and ethical guidelines.
